# Bat rabies surveillance in Finland

**DOI:** 10.1186/1746-6148-9-174

**Published:** 2013-09-08

**Authors:** Tiina Nokireki, Anita Huovilainen, Thomas Lilley, Eeva-Maria Kyheröinen, Christine Ek-Kommonen, Liisa Sihvonen, Miia Jakava-Viljanen

**Affiliations:** 1 Finnish Food Safety Authority Evira, Mustialankatu 3, Helsinki FI-00790, Finland; 2 Section of Biodiversity and Environmental Science, Department of Biology, University of Turku, Turku FI-20014, Finland; 3 Finnish Museum of Natural History, University of Helsinki, P.O. Box 17, Helsinki FI-00014, Finland; 4 Department of Veterinary Biosciences, Faculty of Veterinary Medicine, University of Helsinki, P.O. Box 66, Helsinki FI-00014, Finland; 5 Ministry of Agriculture and Forestry, Food Department, Animal and Plant Health, Mariankatu 23, Helsinki FI-00023, Finland

**Keywords:** EBLV, Lyssavirus, Rabies, Seroprevalence

## Abstract

**Background:**

In 1985, a bat researcher in Finland died of rabies encephalitis caused by European bat lyssavirus type 2 (EBLV-2), but an epidemiological study in 1986 did not reveal EBLV-infected bats. In 2009, an EBLV-2-positive Daubenton’s bat was detected. The EBLV-2 isolate from the human case in 1985 and the isolate from the bat in 2009 were genetically closely related. In order to assess the prevalence of EBLVs in Finnish bat populations and to gain a better understanding of the public health risk that EBLV-infected bats pose, a targeted active surveillance project was initiated.

**Results:**

Altogether, 1156 bats of seven species were examined for lyssaviruses in Finland during a 28–year period (1985–2012), 898 in active surveillance and 258 in passive surveillance, with only one positive finding of EBLV-2 in a Daubenton’s bat in 2009. In 2010–2011, saliva samples from 774 bats of seven species were analyzed for EBLV viral RNA, and sera from 423 bats were analyzed for the presence of bat lyssavirus antibodies. Antibodies were detected in Daubenton’s bats in samples collected from two locations in 2010 and from one location in 2011. All seropositive locations are in close proximity to the place where the EBLV-2 positive Daubenton’s bat was found in 2009. In active surveillance, no EBLV viral RNA was detected.

**Conclusions:**

These data suggest that EBLV-2 may circulate in Finland, even though the seroprevalence is low. Our results indicate that passive surveillance of dead or sick bats is a relevant means examine the occurrence of lyssavirus infection, but the number of bats submitted for laboratory analysis should be higher in order to obtain reliable information on the lyssavirus situation in the country.

## Background

Rabies is a viral zoonosis that causes fatal encephalitis in mammals. The Lyssavirus genus includes 12 recognized species also referred to as genotypes: classical rabies virus (RABV), Lagos bat virus (LBV), Mokola virus (MOKV), Duvenhage virus (DUVV), European bat lyssavirus type 1 (EBLV-1), European bat lyssavirus type 2 (EBLV-2), Australian bat lyssavirus (ABLV), Irkut virus (IRKV), West Caucasian bat virus (WCBV), Khujand virus (KHUV) and Aravan virus (ARAV) [1]. Shimoni bat virus (SHBV) was recently ratified by the International Committee on Virus Taxonomy [2]. Furthermore, novel lyssaviruses have been detected in a Natterer’s bat in Germany, named as Bokeloh Bat Lyssavirus (BBLV) [3], which has subsequently also been found in France [4], and Ikoma Lyssavirus (IKOV) in an African civet (*Civettictis civetta*) [5]. A new tentative lyssavirus, Lleida bat lyssavirus (LLEBV), was found in a bent-winged bat (*Miniopterus schreibersii*) in Spain [6].

Since the first case of bat rabies in Germany in 1954, 1040 cases of rabid bats have been reported in Europe [7]. There have been about 20 cases of EBLV-2, which have occurred in five countries: Switzerland [8], the Netherlands [9], the UK [10,11], Germany [12] and Finland [13]. EBLV-2 is typically detected in Daubenton’s bat (*Myotis daubentonii*) and the pond bat (*M. dasycneme*), whereas the vast majority of EBLV-1 detections have been from the Serotine bat (*Eptesicus serotinus*) [7]. Spill-over infections have been described for EBLV-1 in a stone marten (*Martes foina*) [14], sheep [15] and domestic cats [16]. Selimov et al. reported a human case associated with a bat in Russia in 1985. This virus was later shown to be EBLV-1 [17]. Two cases of EBLV-2 infection in humans, both in individuals working with bats, have been confirmed: one in Finland in 1985 [18] and one in the UK in 2002 [19].

In the UK, EBLV-2 has been isolated from Daubenton’s bats eight times [20]. In 2003, in a targeted surveillance of Daubenton’s bats, a seroprevalence of 0.05% to 3.8% was detected. However, the virus was not detected from the 218 swabs tested [20]. A study from 2003 to 2006 revealed a seroprevalence of 1.0% to 4.1% [21].

In Switzerland, three out of 837 brains examined between 1976 and 2009 were found to be positive for EBLV-2. In targeted active surveillance in 2009, one oral swab from a Daubenton’s bat out of 237 bats swabbed was positive for EBLV-2 and three bats were seropositive [22]. In Germany, 1800 bats were tested between 1997 and 2007, out of which 45 were Daubenton’s bats and one a pond bat. EBLV-2 was isolated in Germany twice, first in 2007 [12] and then again in 2012 [23]. In the Netherlands, 129 suspected pond bats were analyzed and 5 were found to be positive for EBLV-2, resulting in a prevalence of 3.9% [9]. (Table 1). In the area of the Former Soviet Union, more than 3000 bats were analyzed for rabies during 1964–2004 and five lyssavirus species were recorded [24]. Virus-neutralizing antibodies have been found in a number of bat species in several European countries, but because of cross-reactivity, seropositivity cannot be linked to a specific lyssavirus [25]. Eight Daubenton’s bats sampled in 2009 in southern Sweden were seropositive when using EBLV-1 as the antigen [26]. Seropositive Daubenton’s bats have also been found in the UK and Switzerland [25].

**Table 1:** Summary of lyssavirus surveillance focusing on Daubenton’s bats in different European countries

**Country**	**No. of bats in active surveillance (No. of positive bats)**	**No. of Daubenton's bats in active surveillance (No. of positive bats)**	**No. of bats in passive surveillance (No. of positive bats)**	**No. of Daubenton's bats in passive surveillance (No. of positive bats)**	**No. of bats in serosurveillance (No. of positive bats)**	**No. of Daubenton's bats in serosurveillance (No. of positive bats)**	**Reference**
Finland	774 (0)	399 (0)	199 (1)	8 (1)	423 (3 – 9)	268 (3 – 9)	This paper, Jakava-Viljanen et al. 2010[13]
	124 (0)	69 (0)	59 (0)	2 (0)			Hagner et al. 1986[27]
Germany				45 (1)			Freuling et al. 2008 [12]
			~1800 (187)				Müller et al. 2007 [28]
			65 (1)				Freuling et al. 2011 [3]
					98 (5)		Schatz et al. 2013 [25]
Netherlands			3873 (256)	111 (0)			van der Poel et al. 2005 [9]
UK			7457 (6)	144 (6)			Harris et al. 2007 [10]
	766 (0)	439 (0)			660 (5 – 16)	363 (4 – 15)	Harris et al. 2009 [21]
	218 (0)	198 (0)			224 (6 – 18)	198 (6 – 18)	Brookes et al. 2005 [20]
	< 900 (1)	> 900 (1)			> 900	~ 27	SNH, reviewed in Schatz et al. 2012 [23]
Sweden			78 (0)	1 (0)	124 (8)	77 (8)	SVA 2009, reviewed in Schatz et al. 2012 [23]
Switzerland	237 (1)	148 (1)	837 (3)	64 (3)	202 (3)	124 (3)	Megali et al. 2010 [22]

To date, thirteen species of bats have been recorded in Finland out of 45 species present in Europe [29]. All of them, apart from the Northern bat (*Eptesicus nilssonii*), are at the northern-most limit of their range, and most of the species are restricted to southern and central Finland. Only five species are common in Finland; the rest are either very rare or only a few individuals have been recorded so far (Table 2) [30]. The vast majority of the Finnish bats are small, weighing c. 10 g, and insectivorous. Despite an epidemiological study in Finland in 1986 of 183 bats, 124 of which were actively sampled and 59 dead, injured or abnormally behaving [27], and subsequent passive rabies surveillance, rabies in bats was not detected in the country until a case in a Daubenton’s bat was confirmed in autumn 2009. Phylogenetic analysis revealed that the EBLV-2 isolate from the human case in 1985 and the isolate from the bat in 2009 were genetically closely related, demonstrating that EBLV-2 may have been circulating in Finland for years [13].

**Table 2:** **Status and distribution of the bat species recorded in Finland, modified from Kyheröinen *et al*. [**[30]**]**

**Species**	**Distribution/status**
***Nyctalus noctula***	Rare, S Finland, migrant
***Eptesicus nilssonii***	Common, widespread, to S Lapland, some records even further north
***Eptesicus serotinus***	Only a few records, S Finland (first record 2008)
***Vespertilio murinus***	Rare, S Finland, migrant
***Plecotus auritus***	Common, widespread, S & Central Finland, to circa 64° N lat.
***Pipistrellus nathusii***	Restricted, S Finland, migrant/vulnerable
***Pipistrellus pipistrellus***	Rare, S Finland, migrant (first record 2001)
***Pipistrellus pygmaeus***	Rare, S Finland, migrant (first record 2007)
***Myotis nattereri***	Rare, S Finland/ endangered
***Myotis brandtii***	Common, widespread, S & Central Finland, to circa 66° N lat.
***Myotis mystacinus***	Common, widespread, S & Central Finland, to circa 66° N lat.
***Myotis daubentonii***	Common, widespread, S & Central Finland, to 66° N lat.
***Myotis dasycneme***	Only a few records, E Finland (first record 2002)

Status categories for red list species are based on Liukko *et al.*[31].

In this study we assessed the prevalence of EBLVs in Finnish bat populations in order to gain a better understanding of the public health risk that EBLV-infected bats pose. In 2010, a targeted active surveillance project was initiated by the Finnish Food Safety Authority Evira together with the University of Turku and the Finnish Museum of Natural History (University of Helsinki). The active sampling was focused on Southwest Finland, in the area where the EBLV-2 positive bat was found in 2009, and on Uusimaa, in the area where the deceased bat researcher had worked in 1985.

## Results

From the passive surveillance of 258 samples (Table 3), all samples were negative, except for one positive Daubenton’s bat in 2009. This positive case has been reported earlier [13].

**Table 3:** Number of bats tested for rabies in passive surveillance during 1985–2012

**Year**	**Unknown/N.A.**	***Eptesicus nilssonii***	***Myotis daubentonii***	***Myotis mystacinus***	***Myotis brandtii***	***Plecotus auritus***	**In total**	**Number of positive samples**
**2012**	8	16	1	6		1	32	0
**2011**	3	6		4			13	0
**2010**		6	1	1			8	0
**2009**	4	13	3	2		2	24	1
**2008**							0	0
**2007**	1	2					3	0
**2006**		1					1	0
**2005**			1				1	0
**2004**		1		2		1	4	0
**2003**			1				1	0
**2002**		3					3	0
**2001**	1						1	0
**2000**	2						2	0
**1999**		2					2	0
**1998**		9					9	0
**1997**	1						1	0
**1996**	5						5	0
**1995**	5						5	0
**1994**	2						2	0
**1993**	1	1					2	0
**1992**	3	6	1	3		1	14	0
**1991**	5						5	0
**1990**	4	3					7	0
**1989**	3	3					6	0
**1988**	13						13	0
**1987**	35						35	0
**1986**	3	33	2	1	8	14	61	0
**1985**	1						1	0
**In total**	100	105	10	19	8	19	261	1

**Figure 1:**
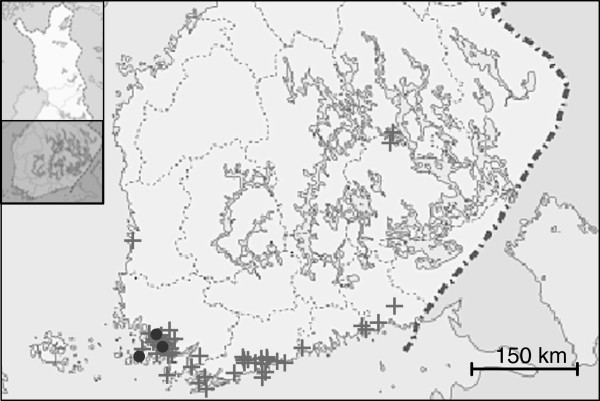
**Map showing the sampling locations of bats in Finland during the active surveillance project in 2010 and 2011 (+).** Seropositive locations are marked with •.

Serum was analyzed from 423 bats of six species in 167 pools (Table 4). Blood samples were collected from 275 and 148 bats in 2010 and 2011, respectively. Antibodies were detected from Daubenton’s bats from two sampling sites in 2010 in the city of Turku (60°27′05″N, 022°16′00″E) and in Nauvo (60°11′35″N, 21°54′25″E), and from one sampling site in 2011 from the city of Naantali (60°28′05″N, 22°01′35″E). All the positive sites were from the same geographical area (Figure 1). The positive samples were from 3 to 9 individuals due to the pooling of samples. All seropositive bats were males. In the pool collected in 2010 from Turku, all individuals were adults and caught while flying. In the pool collected in Nauvo, all bats were adults and they were caught in roosts. In the seropositive pool collected in 2011 from Naantali, one individual was an adult and two were juveniles, and they were caught while flying. Altogether, 268 samples from Daubenton’s bat were analyzed, resulting in a seroprevalence of 1.12% to 3.36%. A 95% confidence interval for the true prevalence of 0.2–4% was calculated, as described by Rogan and Gladen [32]. No EBLV RNA was detected in any of the oropharyngeal swabs analyzed (Table 4).

**Table 4:** Number of bats tested for cross-neutralizing lyssavirus antibodies and viral EBLV-1 and -2 per species and number of positive bats during active sampling in 2010 and 2011

**Species**	**Number of bats (number of positive bats) tested for lyssavirus antibodies**	**Number of bats (number of positive bats) tested for EBLV-1 and -2 RNA**
***M. daubentonii***	268 (3 - 9*)	399 (0)
***M. brandtii***	71 (0)	129 (0)
***P. auritus***	38 (0)	98 (0)
***E. nilssonii***	29 (0)	108 (0)
***M. mystacinus***	16 (0)	36 (0)
***M. nattereri***	1 (0)	1 (0)
***P. nathusii***	0	3 (0)
**Total**	423 (3 - 9*)	774 (0)

* Pooled samples.

## Discussion

A working group of the European research consortium Med-Vet-Net has established guidelines on passive and active bat rabies surveillance. This scheme was also adopted in EUROBATS Resolution 5.2 and in a scientific report submitted to the European Food Safety Authority in 2010 [28,33]. Despite this, the surveillance of bat lyssaviruses varies considerably between European countries, as reviewed by Schatz et al. [25]. In Finland, an active surveillance project was initiated in order to complement passive surveillance after the first EBLV-2 virus detection in a bat in 2009. We demonstrated that EBLV-2 appears to be endemic in Finland in the studied area.

Passive surveillance of bat rabies might be a sufficient and relevant means for obtaining information on its occurrence in Finland. However, the number of bats included in passive surveillance needs to be higher than during 1985–2012, and means to obtain more samples should be considered. In particular, more Daubenton’s bat samples should be tested, as it has been shown that there is a significant correlation between the number of positive bats and the number of bats examined. Thus, it appears that infected bats are more likely to be sampled [34]. Daubenton’s bats do not usually roost in buildings, reducing the likelihood of property owners finding grounded bats. This might lead to lower passive sampling of this species. Specimens of Daubenton’s bats sent by the public to natural history museums and to laboratories are less numerous compared to species such as Brandt’s bats (*M. brandtii*) or Northern bats (*E. nilssonii*), which commonly roost in house attics or other sheltered structures in buildings. It has been demonstrated that most positive samples are detected in the third quarter of the year [25]. In Finland, passive surveillance samples are mostly sent between July and September.

Active sampling of oral swabs has rarely resulted in positive findings, and our findings are consistent with this. Over 900 samples have been tested in Scotland and 148 samples in Switzerland, with only one EBLV-2 viral RNA positive Daubenton’s bat being found in each country. In Spain, 49 *E. isabellinus* samples out of 1297 samples analyzed were positive for EBLV-1 [25]. New lyssaviruses have been found when many bats have been killed for surveillance purposes [24], but invasive sampling is prohibited in Europe according to Council Directive 92/43/EEC on the conservation of natural habitats and of wild fauna and flora (94/43/EEC) and the Agreement on the conservation of populations of European bats.

Serological testing can be used as an indicator of past exposure to lyssavirus in bats. Seropositive Daubenton’s bats have been recorded in the UK, Switzerland, Sweden (reviewed in Schatz et al. 2012 [25]), and now in Finland. Results from our surveillance indicate that the seroprevalence of antibodies against bat lyssaviruses in the Finnish bat population is low. All seropositive sampling sites exhibit high Daubenton’s bat densities. The samples also represent individuals of the same large population, with high frequencies of gene flow between individuals of separate sampling sites [35]. The seroprevalence in bats of antibodies presumably against EBLV-2 has been low, as in our study. However, the seroprevalence of antibodies against RABV in New World bats and against EBLV-1 in Spain have been relatively high (reviewed in Franka et al. 2008 [36]). In an experimental infection, none of the Daubenton’s bats infected with EBLV-2 seroconverted [37]. On the other hand, in an experimental infection of North American big brown bats with EBLV-1, all bats seroconverted after intradermal infection. Seroconversion appears to depend on the viral dose, bat species, route of exposure and the lyssavirus species infecting the bats [36]. The results of serosurveillance are not fully comparable because of differences in methods and antigens used, as well as in threshold values. Using a reciprocal titer of 27 or more as a positive cut-off level may underestimate the actual number of EBLV-seropositive bats, especially when samples have been pooled. Furthermore, reducing the cut-off value of a serological assay results in an increase in sensitivity but a decrease in specificity.

The health risk to the general public, which has no contact with bats through work or leisure, is considered negligible. However, resident bat species in Finland can be infected not only with EBLV-2 but also with other lyssaviruses, including lyssaviruses that have not yet been identified. The most common bat species in Finland is the northern bat (*Eptesicus nilssonii*), which has not been associated with lyssavirus infections. Only a few observations of the species *E. serotinus* have so far been recorded in Finland since the first specimen found in 2008. *Myotis dasycneme* and *M. nattereri* are also very rare in Finland. The distribution ranges of bats in Finland are still poorly known due to the limited number of bat studies carried out in the country.

Positive lyssavirus findings in Finland are from Daubenton’s bat. Daubenton’s bat is a small, widespread and common Eurasian bat species. It is considered a facultative seasonal migrant, covering middle-range distances between summer and winter roosts, often within a distance of 100–150 km [38]. Studies have demonstrated that females, in particular, change their roost relatively often, usually after a few days [39]. Factors that could enhance the maintenance of EBLVs in bat populations include the mobility of many bat species, allowing a high level of gene flow. Laine et al. [35] observed no population structuring in a study of specimens from a broad geographical range in southern Finland and the United Kingdom, Spain and Switzerland. Daubenton’s bats, like many other species of the *Myotis* genus, live in fusion-fission colonies, where all the individual bats in the same roosting area form a loose colony, although no two individuals spend all their time together [40]. The fused colony is formed of a number of subgroups, because it is most likely that an entire colony is comprised of more bats than could fit into a single roost. Fission occurs during roost switching, when subgroups break apart and mix, with the bats ending up in different roosts [41]. This allows bats to expand their social network and effectively increase their colony size without all colony members occupying the same roost. This has many ecological advantages, but also facilitates the effective spread of disease in an extended colony.

## Conclusions

We demonstrated that bat lyssavirus infection appears to be endemic in the studied area in Finland. Passive surveillance of bat rabies might be a sufficient and relevant means for obtaining information on the occurrence of bat rabies in the country. Results from our active surveillance in 2010 and 2011, earlier active surveillance in 1986 and ongoing passive surveillance indicate that the prevalence of EBLV in the Finnish bat population is low. The numbers of bats submitted in passive surveillance should, however, be higher, and means to obtain more samples should be considered. In Finland, the health risk to the general public, which has no contact with bats through work or leisure, is still considered negligible.

## Methods

### Passive surveillance

Dead bats or bats showing clinical signs were submitted by veterinarians, members of the public, animal shelters and bat biologists. The number of bats tested during 1985–2012 was 258, and details are presented in Table 3.

### Active surveillance

#### ***Ethical approval and permission***

The National Animal Experiment Board of the County Administrative Board of Southern Finland approved the sampling of bats, including blood and saliva sampling (permit numbers: Lilley ESLH-YM-2007-01055, Kyheröinen ESLH-2009-04958/Ym-23), which followed Finnish legislation, namely the Finnish Act on the Use of Animals for Experimental Purposes (62/2006). Bats were captured and handled under permits from regional environmental centers (Centre for Economic Development, Transport and the Environment): Southeast region, KASELY/379/20/07.01/2010; Southern Finland, LUO 459/UUS-2009-L-388-254 and UUDELY/475/07.01/2011; Southwest region, LOS-2007-L-182-254; Eastern region, POSELY/501/07.01/2010 and ESAELY/557/07.01/2010.

Active surveillance took place in 2010 and in 2011, most of the bats being sampled for saliva and serum during the summers of 2010 and 2011. Individuals of seven bat species (Table 4) were caught from 71 sampling sites using a combination of harp traps and mist nets placed across woodland corridors, which bats use to commute to foraging areas at dusk, and across foraging sites such as river banks. On some occasions, capture took place near the roost in a building. A Sussex Autobat acoustic lure, placed in front of a harp trap, was used to attract bats to the site [42]. Bats were also caught from bat boxes that had been monitored for several years. Altogether, 190 bats were sampled from roosts and 584 bats were caught while flying.

In some cases, captured bats were fitted with radio transmitters (Holohil LB-2) in order to follow them to the roost. In the case of Daubenton’s bats, the roost was primarily sampled, located in bird boxes or abandoned woodpecker cavities. This enabled the sampling of the whole roost, either directly by taking the bats from the opened nest box or by placing a small mist net in front of the woodpecker cavity opening and waiting for the bats to vacate the roost at dusk. At a single site, Daubenton’s bats were sampled from the ceiling of an old mill.

Care was taken to only handle the bats with gloves thick enough to prevent penetration if bitten. All sampled bats were identified to species, gender was recorded and the age category (juvenile, subadult or adult) was determined from the calcification of the digit joints. Forearms were measured and the individuals were weighed. Saliva and blood samples were subsequently collected. The species distribution and respective sample numbers are presented in Table 4. Most of the samples were taken in southern Finland, in the coastal area ranging from Hamina (near the Russian border) in the east to the Turku Archipelago in the west (Figure 1).

Saliva samples were collected from 459 and 315 bats in 2010 and 2011, respectively. The specimens were collected from the oral cavity and oropharynx using dry nylon fiber- tipped oral swabs (Copan) and transported in a Copan Universal Transport Medium (UTM-RT) System (Copan Diagnostics Inc.). They were kept frozen until laboratory analysis.

Blood samples were collected into a single 75-μl capillary tube from the interfemoral vein after lancing with a 27-gauge needle. The capillary tubes were centrifuged and the hematocrit was measured with a caliper. Serum for rabies antibody screening was stored in Eppendorf tubes, which were kept frozen at -20°C until laboratory analysis.

### Analysis of the samples

Routine rabies laboratory diagnosis was performed on brain samples from passive surveillance according to the OIE Manual of Standards for Diagnostic Tests and Vaccines, 2009 [43], using a standard fluorescent antibody test (FAT). Two conjugates were used: rabies conjugate anti-nucleocapsid (Bio-Rad, USA) and FITC anti-rabies monoclonal globulin (Fujirebio Diagnostics Inc., USA). Conjugates were diluted according to the manufacturers’ instructions.

The RNA for PCR studies was extracted from oral swabs with a QIAamp Viral RNA Mini Kit (Qiagen, Hilden, Germany) according to the manufacturer’s instructions. RNA was tested for EBLV-1 and EBLV-2 with the OneStep real-time reverse transcription-polymerase chain reaction (OneStep RT-PCR kit, Qiagen, Hilden, Germany) test. The reaction volume was 50 μl and the temperature profile of cDNA synthesis and amplification was: 50°C for 30 min, 95°C for 15 min and 45 cycles of 95°C for 30 s, 52°C for 30 s, and 72°C for 20 s. The primer (JW12, N165-146) and the probe (LysGT5 and LysGT6) sequences were published by Wakeley et al. 2005 [44].

Serological testing for EBLV virus neutralizing antibodies was performed using a fluorescent antibody virus neutralization test [45] according to modification (mFAVN) by the UK Animal Health and Veterinary Laboratories Agency (AHVLA). The volume of sera used was 30 μl. The EBLV-2 antigen, strain RV 628, was provided by the European Virus Archive supported by the European Community. Two positive control sera, both provided by the European Virus Archive, were used in each test. Samples often needed to be pooled to obtain the minimum serum volume required for the test. In total, 167 pools were tested. Pooled samples were from animals of the same species, gender and site. Samples were analyzed in duplicate and serially diluted using a 3-fold series. A threshold of 1:27 was chosen to distinguish positive from negative samples.

## Competing interests

The authors declare that they have no competing interests.

## Authors’ contributions

TN participated in the design of the study, set up the rabies serology, analyzed the passive and active survey samples and data and wrote the manuscript. AH participated in the design of the study, set up the rRT-PCR test and participated in manuscript preparation. TL and EMK participated in the design of the study, collected bat samples, participated in discussion of the project and contributed to the manuscript preparation by writing the bat biology and sample collection parts. CEK participated in setting up the rabies serology. LS and MJV designed, coordinated and supervised the study, and participated in data analysis and manuscript preparation. All authors read and approved the final manuscript.
